# Antagonistic effect of alkaloids and saponins on bioactivity in the quinine tree (*Rauvolfia caffra* sond.): further evidence to support biotechnology in traditional medicinal plants

**DOI:** 10.1186/1472-6882-13-285

**Published:** 2013-10-26

**Authors:** Trizah K Milugo, Leonida K Omosa, James O Ochanda, Bethwell O Owuor, Fred A Wamunyokoli, Julius O Oyugi, Joel W Ochieng

**Affiliations:** 1 Center for Biotechnology and Bioinformatics, University of Nairobi, P.O. Box 30197, Nairobi 00100, Kenya; 2 Department of Chemistry, University of Nairobi, P.O. Box 30197, Nairobi 00100, Kenya; 3 Department of Natural Science, Catholic University of Eastern Africa, P.O. Box 62157, Nairobi 00200, Kenya; 4 Department of Biochemistry, Jomo Kenyatta University of Agriculture and Technology, P.O. Box 62000, Nairobi 00200, Kenya; 5 Department of Medical Microbiology, College of Health Sciences, University of Nairobi, P.O. Box 19676, Nairobi 00202, Kenya; 6 College of Agriculture & Vet Sciences, University of Nairobi, P.O. Box 30197, Nairobi 00100, Kenya

**Keywords:** *Rauvolfia caffra*, Antioxidant, Alkaloid, Quinine tree

## Abstract

**Background:**

The Quinine tree (*Rauvolfia caffra*) is used as a medicinal plant among traditional communities in many countries to manage tumors and other diseases associated with oxidative stress. To validate indigenous knowledge and possibly position this herb for technology uptake and utilization, we established the level of antioxidant activity in *R. caffra*, and probed for the presence of associated phytochemicals.

**Methods:**

Antioxidant activity was determined on 1,1-diphenyl-2-picrylhydrazyl (DPPH) while major phytochemicals were identified by multiple tests on methanol fractions.

**Results:**

*R. caffra* showed promise as a cure, with antioxidant activity comparable to the commercially used drug quercetin (*R. caffra* = 79.7% ±1.9; quercetin = 82.6% ± 2.0). However, we found two phytochemicals with possible antagonistic effect: co-occurrence of alkaloids and saponins significantly reduced antioxidant activity (alkaloids only = 63%; alkaloids plus saponins = 15%; steroids, terpenoids and cardiac glycosides = 82%), thus alkaloids and saponins should be exclusive to each other in drug formulations.

**Conclusions:**

Antagonistic relationship among phytochemicals would affect the efficacy of crude extracts as used in traditional medicine. Unlike in herbal medicine, use of modern biotechnology in extraction, purification and design of optimal combinations will ensure efficient drug formulations with optimum bioactivity and minimum toxicity. Metabolic pathway engineering under a controlled environment may optimize availability of desired compounds.

## Background

Medicinal plants are widely used in many traditional cultures and are increasingly becoming popular in modern society as natural alternatives to synthetic medicines. Traditional herbs are generally cheaper, accessible or readily available and more culturally acceptable to many. Further, some synthetic drugs have been suspected to cause side effects [1,2], thereby making many turn to traditional herbs as complementary therapies and for preventive medicine. Lately, there has been a global upsurge in the incidences of cancer, hypertension and other diseases related to oxidative stress especially in developing countries. Globally, herbal medicine is gaining popularity even in regions with improved healthcare systems [3]. Medicinal plants are used in reducing predisposition to, and managing diseases related to oxidative stress, such as cancer and hypertension, because they contain strong antioxidants such as flavonoids/anthocyanins and alkaloids. Indeed, anthocyanins have been used in therapy against cardiovascular diseases and cancer [4] and age related conditions such as Alzheimer’s disease or dementia [5].

Although medicinal plants have been exploited by traditional societies to manage certain ailments, the traditional prescription of crude extracts is unsuitable for two reasons: first, some phytochemicals may occur at toxic levels in crude extracts [6] and the herbalists or patients have no mechanism to determine the level of toxicity when whole extracts are administered, and second, the bioactivity may be suboptimal because maximum activity requires certain combinations of phytochemicals. In addition, the doses often are usually taken repeatedly and exceed conventional drugs in volume. Further, genetic variation within a plant species may correspond to variation in phytochemical composition, thus affecting the levels of abundance for the bioactive compounds [7]. The difficulty in predicting the phytochemical composition, which may vary significantly and which due to the variation may be taken at toxic levels, makes traditional herbs unsuitable as crude extract as currently practiced. Exploitation of medicinal plants using modern biotechnology in purification and separation of compounds, and in metabolic engineering where relative abundance of various phytochemicals can be influenced would result in optimum activity and safety. With new developments in genetic engineering, production of compounds known to have important bioactivity related to human health such as antioxidant action, anti-inflammatory and antimicrobial effects can now be influenced for continuous supply of rare and expensive secondary metabolites.

Use of modern biotechnology, especially genetic engineering, remains under sustainable resistance from many communities and interest groups. Although genetic manipulation (GM) is a controversial subject, stronger objection usually concerns food crops, where GM activities are feared to affect unintended human populations. To avoid this controversy and to respect consumer choices, it is necessary that genetic engineering for desired phytochemicals with health benefits target non-food crops. Further, the plant species of choice must have properties that guarantee that GM activities on the plant do not cause environmentally unacceptable outcomes such as genetic pollution.

Rauvolfia is a genus of evergreen trees and shrubs in the dogbane family, Apocynaceae. Quinine tree (*Rauvolfia caffra*) is used as a medicinal plant among traditional communities in many countries to manage tumors and other diseases associated with oxidative stress [8]. This species is still of less conservation concern in many countries, such as South Africa [9]. However, another member of this genus, *Rauvolfia serpentina*, is now declining in the wild due to over-exploitation for its medicinal uses and is currently listed in CITES Appendix II [10]. Genetic modification would be less controversial for *R. caffra* since it is not a food crop but a forest tree. Although *R. caffra* is a sparsely populated remnant tree species that is purely selfing, hence the threats of gene flow is limited, it should be domesticated under controlled conditions where the bioactive molecules can then be extracted and used for medicinal purpose.

This study aimed to identify available phytochemicals in *R. caffra* with known function in human health, and to test individual and collective bioactivity of these compounds. Two phytochemicals, alkaloids and saponins, were found to have possible antagonistic effect, where their co-occurrence significantly reduced antioxidant activity in *R. caffra*, thus alkaloids and saponins should be exclusive to each other in drug formulations.

## Methods

### Sample collection, storage and treatment

This study utilized tissue samples of quinine tree (*R. caffra*) from a remnant forest in Kuria District of Western Kenya. A mature *R. caffra* tree was identified with the assistance of a traditional medicine practitioner. In March 2013, terminal leaves were collected from the lower branches while bark samples were collected by chopping from the trunk. Upon collection, samples were kept in air-tight bags with silica gel as desiccant and transported to the laboratory for processing. Samples were authenticated by a botanist, Professor J.O. Kokwaro. Herbarium specimens were deposited at the University of Nairobi herbarium, under specimen Voucher No: Mutiso-RC-23/3/2012.

The leaves and stem bark were air dried at room temperature under shade for one week, ground into powder and subjected to extraction using organic solvents in the order of polarity: Hexane, then Dichloromethane (DCM) and finally, Methanol using standard procedures [11] to obtain crude extract. Extracts were weighed before further processing.

### Thin Layer Chromatography (TLC) assay

Thin Layer Chromatography is a method used to separate compounds based on their solubility in organic solvents and molecular weight. This method was used to detect the presence of compounds in the crude extracts. The crude extracts were spotted on a TLC plate and developed first in pure DCM, followed by DCM/Methanol (97:3 v/v) and finally DCM/Methanol (19:1). The TLC plates were then air dried and compounds detected under UV light. To detect samples with antioxidant activity, the plates were sprayed with DPPH (1, 1-diphenyl-2-picrylhydrazyl; 12 mg DPPH in 50 ml double distilled methanol) and incubated for 30 minutes at room temperature as described previously [12]. The presence of whitish spots against a purple background on the plate would be considered to indicate radical scavenging properties. Such extracts were selected for further analysis using DPPH method.

### DPPH free-radical scavenging assay

Samples indicating antioxidant activity were analyzed further using DPPH assay. 200 μg of leaves and stem bark crude extracts were dissolved in double distilled methanol and serially diluted to concentrations ranging from 6.3 μg/ml to 200 μg /ml. 0.5 ml of each dilution was added to 3 ml of 0.1 mM DPPH and incubated for 30 mins at room temperature [13]. The radical scavenging activity was determined using UV light absorbance method, measured at 517 nM. A decrease in optical density (OD) indicated radical scavenging activity in the sample. Commercially available quercetin (3,3′,4′,5,7-Penthydroxyflavone) was used as a positive control, and methanol as negative control.

Statistical analysis:

The percentage inhibition of free radical formation (%) was calculated as follows.

$$\frac{\text{Inhibition}\;\%=\left(A_{\text{blank}}-A_{\text{sample}}\right)}{A_{\text{blank}}},$$

Where: A_blank_ is the absorbance of the negative control, while A_sample_ is the absorbance of the test sample.

The Means of inhibition percentages, their standard deviations and a test of whether the means were statistically different from each other was calculated using EXCEL, via a student t test with significance level set at p ≥ 0.05.

### Fractionation of phytochemicals in stem bark crude extract

Stem bark methanol crude extract (15 g) was re-dissolved in a combination of DCM:n-hexane (5% DCM) and successively separated with solvent combination of DCM: n-hexane as the starting solvent, followed by solvent combinations of increased polarity (DCM increased to 10%, 15%, 20%, 30%, to 100%; then DCM/methanol, with methanol increased to 0.5%, 1%, 2%, to 10% by volume), resulting into a total of 120 fractions (50 ml each). Aliquots of the fractions were spotted on TLC plates and developed using various solvent systems, including DCM: n-Hexane; 100% DCM; 1% Methanol: DCM; 5% Methanol: DCM; 8% Methanol: DCM. Fractions with the same characteristics were pooled and concentrated to dryness on a rotary evaporator giving a total of 15 fractions. Identification of chemical groups followed standard procedures described previously [14,15]. The phytochemicals probed were: flavonoids, coumarins, alkaloids, steroids, cardiac glycosides, saponin, terpenoids, tannins and phenols.

## Results and discussion

This study sought to establish whether phytochemicals of known function in human health are found in quinine tree (*Rauvolfia caffra*), a plant used as medicine among traditional communities in many countries to manage tumors and other diseases associated with oxidative stress. The presence of such phytochemicals would validate indigenous knowledge held by traditional communities regarding its medicinal value, and position quinine tree as candidate for exploitation using modern biotechnology. We found *R. caffra* to be rich in antioxidants, and contain several known phytochemicals, two of which showed antagonistic effect.

*Plant ecology and description*: Quinine trees were confirmed to grow in the localities that were earlier described by traditional practitioners in Kuria, Western Kenya. We found three isolated trees growing near a valley with a stream running about 500 metres from the tree. The leaves were shinny green on the upper surface, suggesting the presence of wax on the epidermal layer. Flowers had both male and female reproductive features, with anthers appearing above the stigmas.

### Quinine tree as a rich antioxidant

All tests showed Methanol used as a negative control to have 0.00% free radical Inhibition, while the commercially available quercetin used as a positive control inhibited within expected levels (82.63% ± 2.00), lending credence to the method as a reliable test for antioxidant activity. Extracts from stem bark samples of *R. caffra* had a free radical Inhibition of 79.65% ± 1.86, while the leaves showed 70.55% ±1.26 (Table 1). There was no statistically significant difference between the free radical scavenging activity of *R. caffra* stem bark extracts and the standard quercetin (p > 0.05), suggesting *R. caffra* to be a competitively strong antioxidant. Indeed, it is likely that *R. caffra* antioxidant activity is stronger than quercetin, given that the samples assayed in this study were crude extracts, while the commercial product is usually a purified compound.

**Table 1:** **Level of free radical inhibition by extracts from *R. caffra *as accessed on DPPH**

**Extract source**	**Free radical inhibition (%)**
Leaves	70.55 ±1.26
Stem bark	79.65 ± 1.86
Commercial quercetin (positive control)	82.63 ± 2.00
**Methanol (negative control)**	**0.00 ± 0.00**

Commercially available quercetin was used as control.

Inhibition values (%) are the means of triplicate measurement (n = 3) ± STD.

The observation of higher antioxidant activity in the stem bark extracts compared to leaves was consistent with previous findings [16] that assayed the plant for antimicrobial activity. Why the stem bark extracts are a better antioxidant is not clear, but can possibly relate to plant storage system or the presence of antagonistic polyphenol(s) in the leaves.

### Phytochemical composition of *R. caffra* crude extract

Screening for phytochemicals in crude extracts from leaves and stem bark of *R. caffra* revealed the following classes of compounds: alkaloids, terpenoids, saponin, cardiac glycosides and steroids (Table 2). These results were consistent for each class of compound even when different tests/approaches were used, albeit with subtle differences in abundance. The detection of alkaloids, terpenoids, saponin, cardiac glycosides and steroids confirmed that *R. caffra* samples indeed contained molecules known for antioxidant activity. These findings reaffirm the value of indigenous knowledge in identification of plants for pharmaceutical use. The use of quinine tree in traditional medicine is validated by presence of phytochemicals of known health benefits. The presence of cardiac glycosides in *R. caffra* may explain why the herb is used traditionally to manage heart diseases.

**Table 2:** **Phytochemicals found in methanol extracts of leaves and stem bark *of R. caffra***

**Compound**	**Test**	**Abundance**	
		**Leaves**	**Stem bark**
**Alkaloids**	Wagnes test	3	3
**Terpenoids**	*p*-anisaldehyde	1	3
	Salkowski test	2	3
**Saponin**	Vanilin/sulphuric acid	1	2
	Foam test	2	2
**Steroids** **Cardiac glycosides**	Chlorofoam/sulphuric acid	2	2
	Keller-Killani test	2	2
**Flavonoids**	HCL-Mg reaction	0	0
	AlCl_3_ reaction	0	0
	Ammonia test	0	0
**Coumarin**	Open loop – close loop response	0	0
**Phenols and tannins**	FeCl_3_ test	0	0
	Vanillin- HCL reaction	0	0

Legend; 3 – Abundant; 2 - Moderately present; 1 - Weakly present; 0 - Absent.

Based on plant physiology, *R. caffra* and similar trees should contain phenols and tannins. Surprisingly, these were not detected even using multiple tests. It is possible that most of the polyphenols were lost in the solvent system (hexane and DCM), before the extracts were subjected to subsequent analysis on methanol. *R. caffra* had leaves which were shiny on the upper surface. Wax which is responsible for the glossy appearance of leaves usually contains phytochemicals including flavonoids, phenolics and sterols.

### Antagonistic bioactivity of alkaloids and saponins

Although crude extracts from stem bark and leaf samples of *R. caffra* showed antioxidant activity (free radical Inhibition) of 79% and 70% respectively (Table 1), analysis of fractions showed activity to vary with phytochemical composition. Strongest antioxidant activity was observed for fractions containing alkaloids, steroids, terpenoids, cardiac glycosides, but without saponins (82.39%; Table 3). Fractions that included saponins, i.e., alkaloids, steroids, terpenoids, cardiac glycosides, saponins had a lowered activity of 58.99%. Alkaloids only had 63% activity, but fractions containing a combination of alkaloids and saponins exhibited the poorest antioxidant activity of 15% (Table 3). Alkaloids and saponins appeared to have antagonistic interaction, at least with regards to antioxidant activity. This potentially lowers their activity as antioxidants, and possibly the potency of extracts containing both compounds. Saponins observed in this study are likely to be steroids, the type common in wild plants used as herbs, while those that occur in cultivated crops are predominantly the triterpenoid saponins [17].

**Table 3:** Phytochemical screening and antioxidant activity of stem bark fractions

**Fraction**	**Alkaloids**	**Steroids**	**Cardiac glycosides**	**Saponins**	**Terpenoids**	**% inhibition**
**F**_**A **_**5-10% HEX/DCM**	**+**	**+**	**+**	**+**	**-**	41.82 ± 3.3
**F**_**B**_**&**_**C **_**10-20% HEX/DCM**	**+**	**-**	**-**	**+**	**-**	15.68 ± 2.2
**F**_**D**_**&**_**E **_**30-40% HEX/DCM**	**+**	**-**	**-**	**-**	**-**	*
**F**_**F **_**50-60% HEX/DCM**	**+**	**-**	**-**	**-**	**-**	62.99 ± 3.7
**F**_**G **_**70–80% HEX/DCM**	**+**	**+**	**+**	**-**	**-**	43.8 ±2.4
**F**_**H**_**&**_**I **_**90-100% HEX/DCM**	**+**	**-**	**-**	**-**	**-**	*
**F**_**J **_**0.5-1% MEOH/DCM**	**+**	**-**	**-**	**-**	**-**	*
**F**_**K**_**&**_**L **_**1-5% MEOH/DCM**	**+**	**+**	**+**	**-**	**+**	38.18 ± 3.6
**F**_**M **_**5-7% MEOH/DCM**	**+**	**+**	**+**	**-**	**+**	82.39 ± 1.4
**F**_**N **_**7-9% MEOH/DCM**	**+**	**+**	**+**	**+**	**+**	58.99 ± 1.9
**F**_**O **_**9-15% MEOH/DCM**	**+**	**+**	**+**	**+**	**+**	*
**F**_**P **_**100% MEOH**	**-**	**+**	**+**	**-**	**+**	*

Legend; + present; - absent * not determined; HEX- Hexane.

### Modern biotechnology and the optimization of medicinal value of *R. caffra*

Traditional practitioners administer tissues from quinine tree as crude extracts to their patients. In this study, we show antagonistic relationship among two phytochemicals (alkaloids and saponins), an observation that can only be obvious when the compounds are fractionated and tested separately. Such antagonism, perhaps involving more compounds than observed in this study, would affect the efficacy of crude extracts as used in traditional medicine. The data suggested that for pharmaceutical purposes, alkaloids and saponins should be exclusive to each other in drug formulations. Further, administration of crude extracts does not take into account the possibility that some compounds could exist at toxic levels. For example, saponins are known to have a lytic action on erythrocyte membranes, a haemolytic action believed to be the result of the affinity of the aglycone moiety for membrane sterols, particularly cholesterol [18], with which they form insoluble complexes [19]. Further, dietary saponins have been reported to obstruct the absorption of micronutrients and to reduce protein digestibility probably by the formation of sparingly digestible saponin–protein complexes [20]. However, on the positive side, it has been demonstrated that saponins affect nutrient uptake through the intestinal membrane, by increasing the permeability of intestinal mucosal cells in vitro, inhibiting active mucosal transport and facilitating uptake of substances that are normally not absorbed [21].

*Genetic engineering of metabolic pathways*: Unlike in herbal medicine, use of modern biotechnology in extraction, purification and design of optimal combinations will ensure efficient drug formulations with optimum bioactivity. Although genetic manipulation is a controversial subject worldwide, stronger objection concerns food crops, where GM activities would affect unintended human populations. Manipulation of wild tree species would only be opposed on the basis of possible gene flow to non target closely related tree species. *R. caffra* is a sparsely populated remnant tree species that is purely selfing, hence the threats of gene flow is limited.

*Variation in phytochemical composition*: *R. caffra* is a self-fertilized plant and would ordinarily be expected to have low levels of genetic diversity. However, we suspect *R. caffra* to have high levels of genetic differentiation, following *R. serpentina*, an equally selfing tree species in the same genus and which have been reported to exhibit high levels of diversity [22]. Genetic variation among plant species may correspond to variation in phytochemical composition, thus affecting the levels of abundance for the bioactive compounds. The difficulty in predicting the phytochemical composition, which may vary significantly, and which due to the variation may be taken at toxic levels, makes *R. caffra* unsuitable as crude extract as currently practiced. This herb would best be exploited using modern biotechnology, where phytochemical composition can be quantified and controlled. With new developments in genetic engineering, phytochemicals known to have important biological activities related to human health such as antioxidant action, anti-inflammatory and antimicrobial effects can now be influenced for continuous supply of rare and expensive secondary metabolites. Metabolic engineering aims at modifying cellular metabolite composition, so that new compounds can be produced, existing compounds can be increased, and/or eliminate undesirable compounds. This can be achieved by introducing novel genes or pathways, and/or enhancing or eliminating the expression of endogenous pathways.

## Conclusion

This study has shown that the use of *R. caffra* by traditional healers has a scientific basis as evidenced by the presence of phytochemicals of known health benefits. However, traditional prescription of crude extracts is unsuitable for two reasons: first, biosafety concern – some phytochemicals may occur at toxic levels in crude extracts and there is no way to determine this when whole extracts are administered, and second, the bioactivity may be suboptimal due to presence of some phytochemicals with antagonistic effects as observed in this study. The plant should best be exploited using modern biotechnology in purification and separation of compounds, and in metabolic engineering under controlled environment where relative abundance of various phytochemicals can be influenced.

## Competing interests

None of the authors has competing interest.

## Authors’ contributions

TKM – designed experiment, collected samples, conducted laboratory analyses, analyzed data, participated in write-up of manuscript. LKO – participated in analysis and reviewed drafts of the manuscript. BOO – collected samples and reviewed drafts of the manuscript. FAW – reviewed drafts of the manuscript. JO Oyugi – data collection and reviewed drafts of the manuscript. JO Ochanda – participated in the design and coordination of the study. JWO – data interpretation and write-up of manuscript. All authors read and approved the final manuscript.

## Pre-publication history

The pre-publication history for this paper can be accessed here:

http://www.biomedcentral.com/1472-6882/13/285/prepub
